# Nutritional Profile and Sensory Acceptance of Sourdough Breads Enriched With Ora‐pro‐nóbis Leaf Powder

**DOI:** 10.1111/1750-3841.70629

**Published:** 2025-10-24

**Authors:** Dayane Lilian Gallani Silva, Filipe Andrich, Barbara Daniele Almeida Porciuncula, Marcelo Augusto Batista, Marcela Moreira Terhaag, Beatriz Cervejeira Bolanho Barros

**Affiliations:** ^1^ Department of Technology State University of Maringá Umuarama Paraná Brazil; ^2^ Federal Institute of Paraná Umuarama Paraná Brazil; ^3^ Department of Agronomy State University of Maringá Maringá Paraná Brazill

**Keywords:** antioxidants, calcium, dietary fibers, instrumental texture, Pereskia aculeata, phenolic compounds

## Abstract

This study aimed to characterize the composition of ora‐pro‐nóbis leaf powder (OPNP) and evaluate the nutritional profile of enriched sourdough breads (SBs). Four SB formulations were prepared using different concentrations of OPNP (SBC, 0; SB1, 1%; SB3, 3%; and SB5, 5%) and a leavening agent (a mixture of lactic acid bacteria and yeast). SBs were evaluated for physicochemical, antioxidant, and sensory properties. OPNP was shown to contain 21.01 g 100 g^−1^ proteins, 30.92 g 100 g^−1^ dietary fiber, 3.28 g 100 g^−1^ calcium, and 69.42 mg 100 g^−1^ total phenolic compounds. Seven phenolic acids and six flavonoids were identified in OPNP. The leavening agent used in SBs production contained 7.40 log colony‐forming units (CFU) g^−1^ lactic acid bacteria and 4.98 log CFU g^−1^ yeasts. SB5 had higher contents of proteins (10%) and dietary fibers (90%) than SBC. There was a gradual increase in calcium, magnesium, phenolics, and antioxidant activity as OPNP concentration increased. Enriched SBs were darker and greener than SBC. By contrast, hardness, elasticity, and chewiness did not differ significantly between formulations. In a hedonic test conducted with consumers, enriched SBs received similar scores for aroma, flavor, and texture. This study presents a sustainable strategy for incorporating non‐conventional food plants into bakery products, with SB3 being the most recommended formulation, owing to its superior nutritional profile compared with SBC and SB1 and higher purchase intention than SB5.

## 1 Introduction

Bread is widely consumed worldwide. In 2021, the health and wellness baked goods sector was valued at US$42.3 billion, with Brazil ranking as the second‐largest market, behind the United States only (Olakanmi et al. 2024). The global sourdough bread (SB) market size was estimated at US$2.83 billion in 2023 and is projected to grow at a compound annual growth rate of 10% through 2032 (Singh 2024). Bread production via sourdough fermentation has become increasingly popular in recent decades. This technique involves the use of lactic acid bacteria (LAB) and/or yeasts, whose actions improve nutritional properties by increasing the bioavailability of minerals (Lopez et al. 2003) and phenolics (Anson et al. 2011), enhancing protein digestibility (Mastrolonardo et al. 2024), and reducing the glycemic index (Scazzina et al. 2009). Sourdough metabolites also contribute to acidification, positively influencing the flavor, texture, and shelf life of bread products (Lafuente et al. 2024).

Another interesting strategy for improving the nutritional profile of staple foods, such as breads, is fortification with plant‐based ingredients and by‐products with high nutritional value (Mastrolonardo et al. 2024). Non‐conventional food plants can serve as sources of dietary fibers and bioactive compounds in bread products, offering health benefits for consumers and addressing the demand for nutritionally enriched foods (Peñalver and Nieto 2024).

An example is *Pereskia aculeata* Miller, a plant occurring in the Brazilian Atlantic Forest and commonly known as ora‐pro‐nóbis (OPN). It is easy to cultivate, with an estimated leaf yield of 2000 kg ha^−1^, requiring low investments from farmers (Possobam and Stroparo 2022). OPNP leaves are often described as a “super food” due to their high levels of dietary fiber (42 g 100 g^−1^ dry basis, d.b.), protein (12–25 g 100 g^−1^ d.b.), calcium (494 mg 100 g^−1^ wet basis, wb), potassium (482 mg 100 g^−1^ wb), magnesium (131 mg 100 g^−1^ wb), and iron (1 mg 100 g^−1^ wb) (Fioroto et al. 2024; Barreira et al. 2020; Sato et al. 2018). The leaves are also rich in phenolic compounds (23.75 mg g^−1^), mainly phenolic acids such as gallic, chlorogenic, caffeic, ferulic, and protocatechuic acids and flavonoids such as quercetin, rutin, and catechin (Fioroto et al. 2024; Garcia et al. 2019). The antioxidant activity of phenolic compounds contributes to increasing the shelf life of food products. Additionally, phenolics play a role in protecting against various diseases and slowing premature aging in humans (Sousa et al. 2014; Cruz et al. 2021).

Given its valuable nutritional profile, OPN has been used in the production of diverse food products, such as pasta (Rocha et al. 2008), sausages (Sobrinho et al. 2015), beverages (Vieira et al. 2020), and *petit‐suisse* (Silva et al. 2021). However, to date, no study has investigated its addition to SBs. Processing the leaves into a powder facilitates their incorporation into food matrices and helps prevent losses associated with their high perishability. Silva et al. (2024) showed that convective drying of OPN leaves at 100°C for 2 h preserves phenolic compounds, yielding a powder with good functional properties. The incorporation of OPN leaf powder (OPNP) into SBs is innovative, particularly when combined with the mixture of whole and white wheat flours to promote the nutritional value and a leavening agent, whose microbiota can provide functional benefits (Silva et al. 2025). In light of these considerations, this study aimed to characterize OPNP composition and evaluate the nutritional and sensory profile of SBs enriched with OPNP.

## 2 Materials and Methods

### 2.1 Raw Materials and Processing

#### 2.1.1 Ora‐pro‐nóbis Leaf Powder (OPNP)

OPN (*P. aculeata*) leaves were collected in the winter season (August 2023) in a medicinal plant farm located in Umuarama/Paraná/Brazil (‐23.7921294, ‐53.255574). Whole OPN leaves were washed in running water and dried in an air‐circulating oven (Nova Ética, Brazil) at 100°C for 120 min (constant weight), as defined by Silva et al. (2024). The dried leaves were crushed in an analytical mill (Ika, USA), and the resulting powder was sieved (0.15 mm) to obtain OPNP.

#### 2.1.2 Leavening Agent

For leavening agent preparation, ripe star fruits (0.96% ± 0.03% acidity, 6.27 ± 0.25 °Bx) were obtained from a local producer, washed, chopped, mixed with water at a ratio of 1:2 (w/w), and incubated at 27°C for 48 h (Limatec BOD chamber, Brazil). Then, part of the fermented liquid (50 g) was collected and mixed with whole wheat flour (25 g) and white wheat flour (25 g), both from Biorgânica Ltda. The mixture was incubated at 27°C for 24 h. Part of this material was mixed with water (boiled and filtered) and wheat flour (half white and half whole grain) at a ratio of 1:1:1 (w/w/w) and incubated at 27°C for a further 24 h. This procedure was repeated daily for 15 days, using the previous day's fermented material as starting material. After the 15‐day period, the resulting leavening agent was stored under refrigeration (4°C). The leavening agent was renewed every 3 days by using the same proportion of ingredients and incubating at 27°C for 3.5 h. This procedure was carried out for 6 months before the leavening agent was used in SB production (Silva et al. 2025).

For analysis, the leavening agent (25 g) was homogenized with peptone water (225 mL) and subjected to sequential dilutions. The material was analyzed according to the methods of the American Public Health Association (APHA 2001). LAB counts were performed by deep plating using De Man–Rogosa–Sharpe (MRS) agar, and the results are expressed in colony‐forming units (CFU) mL^−1^. Acidified potato dextrose agar was used for yeast counting, and the results are expressed in CFU mL^−1^.

#### 2.1.3 Sourdough Bread Formulations

Four SB formulations were prepared by varying OPNP concentration: 0 (control, SBC), 1% (SB1), 3% (SB3), and 5% (SB5). In the autolysis stage, a mixture of white and whole wheat flour (Biorgânica Ltda), sugar (Native Organic Products), and water was used, according to Table 1S. The proportion of ingredients was based on preliminary tests. A mixture of white and whole wheat flours was used to enhance the nutrient content of SBs, with adequate gluten network formation.

The mixture of ingredients was maintained at 28°C for 60 min. Then, the leavening agent, previously fed with flour and water, was added to the mixture. The resulting dough was kneaded in a planetary mixer (Oster, USA) for 5 min. Salt (Temperos Al‐Andalus) was added, and the dough was kneaded for another 5 min. The dough was then left to rest for 10 min, and four folds were made with a 30 min interval between them. In the bread‐shaping stage, the dough was flattened and smoothed to expel part of the carbon dioxide produced in the first fermentation. The dough was then folded from the edges to the center, ensuring that the joints were well consolidated so that the bread would not open during baking. After the second fermentation, the dough was placed on a glass pan (Ô Cuisini) and baked in an oven (Progás, Brazil) at 220°C for 12 min, 210°C for 6 min, and 190°C for 12 min. After cooling at 28°C, the breads were cut into slices approximately 1.5 cm thick and stored in hermetically sealed plastic Ziplock bags (Silva et al. 2025).

### 2.2 Analytical Methods

#### 2.2.1 Chemicals

Folin‐Ciocalteau, DPPH (2,2‐diphenyl‐1‐picrylhydrazyl), Trolox (6‐hydroxy‐2,5,7,8‐tetramethylchroman‐2‐carboxylic acid), methanol, gallic acid, and trimethylchlorosilane were purchased from Sigma‐Aldrich. Sodium carbonate, ethanol, sodium hydroxide, sodium chloride, hydrochloric acid, and zinc sulfate were purchased from Anhydrol. The other reagents used were ferric chloride (Dinâmica) and n‐hexane (Química Moderna).

#### 2.2.2 Proximate Composition and Mineral Analysis

Proximate composition was assessed using the official methodology of the Association of Official Analytical Chemists (AOAC 2019): moisture (method 925.09), ashes (method 923.03), proteins (method 920.87), crude fat (method 920.85), and total dietary fiber (TDF), soluble dietary fiber (SDF), and insoluble dietary fiber (IDF) (method 985.29). The carbohydrate content was estimated by the difference in the determinations made for moisture, ash, proteins, crude fat, and TDF.

Samples were subjected to nitro perchloric digestion (90°C, 2 h), followed by the analysis of calcium (Ca), copper (Cu), iron (Fe), potassium (K), magnesium (Mg), manganese (Mn), phosphorus (P), and zinc (Zn) by microwave plasma atomic emission spectrometry (MP‐AES) using a Microwave Plasma Atomic Emission Spectrometer (MP‐AES 4200, Series 4200), Agilent (Santa Clara, CA, USA) (Besen et al. 2024).

#### 2.2.3 Extraction and Analysis of Antioxidant Compounds

To evaluate phenolic compounds and antioxidant activity, OPNP and SB formulations were subjected to extraction with a hydroalcoholic solvent (70% v/v ethanol, 30% v/v distilled water) in a ratio of 1:20 (m/v). The mixture was shaken at 130 rpm for 2 h at 25°C, and the supernatant was used in the analysis.

Th phenolic composition of OPNP extract was evaluated by ultra‐high performance liquid chromatography (UHPLC, Shimadzu Nexera X2, Kyoto, Japan) equipped with a mass spectrometer (MS, Bruker IMPACT II, Billerica, MA, United States), an electrospray ionization (ESI) source, a quadrupole‐time‐of‐flight (Q‐TOF) analyzer, and a multichannel plate (MCP) detector. The capillary voltage was operated in positive and negative ionization mode, set at 4500 V, and with an endplate displacement potential of −500 V. Gas parameters were set to 8 L min^−1^ at 200°C and fragmentation was performed using argon collision gas (5–45 eV). Data were collected from m/z 50–1950 at a resolution of 50,000 and with an acquisition rate of 5 spectra per second. Ions of interest were selected by automatic scanning fragmentation MS/MS. Chromatographic separation was performed using a C18 column (75 × 2.0 mm i.d.; 1.6 µm Shim‐pack XR‐ODS III), using a gradient mixture of solvents A (ultrapure water) and B (acetonitrile) as follows: 5% B 0–2 min, 30% B 2–3 min, 95% B 3–12 min, maintained at 95% B 12–15 min, and 5% B 15–16 min, at 40°C. The data processing software was Data Analysis 4.3 (Bruker). The identification of the compounds was proposed based on a literature review of the genus and family of study and of common chemical classes in plants, in addition to the mass error value (Brenton and Godfrey 2010). The UHPLC system is equipped only for qualitative analysis.

The content of total phenolic compounds (TPC) was determined by a spectrophotometer (Shimadzu, model 1900, Kyoto, Japan) according to Singleton et al. (1999). Analytical curves (*R* ^2^ > 0.99) were prepared with gallic acid solutions (25 to 500 mg L^−1^). The results were expressed as gallic acid equivalent (GAE).

The antioxidant capacity by the DPPH method was performed according to Blois (1958). The iron reducing antioxidant power (FRAP) methodology was used as described by Benzie and Strain (1996). For each method, an analytical curve was constructed with Trolox solutions (0.10 to 1.00 mM), and the results were expressed in Trolox equivalent (TE).

#### 2.2.4 Instrumental Color

The color parameters, L* (lightness), a* (greenness–redness), and b* (blueness–yellowness), were evaluated in OPNP and SB formulations. This analysis was carried out on a colorimeter (CR‐10, Konica Minolta, Tokyo, Japan), using the CIELab system (CIE 2019).

#### 2.2.5 Functional Properties

The functional properties of OPNP were investigated. Water absorption index (WAI) was assessed by stirring 0.5 g of sample with distilled water (10 mL) at 200 rpm for 3 h in an orbital shaker (Tecnal TE‐141). After centrifugation, WAI was calculated by the ratio of wet sediment weight to sample weight. The supernatant was dried at 105°C until constant weight (oven Marconi MA 035/3BX) to obtain the water solubility index (WSI). The WAI method was repeated, replacing water with soybean oil, to estimate the oil absorption index (OAI) (Meram and Tontul 2024).

#### 2.2.6 Texture Profile and Acidity

Texture profile analysis (TPA) was determined in a texturometer (TA‐XT2i, Extralab) equipped with a cylindrical probe of 7 cm of diameter, moving at 1.00 mm s^−1^ and a load cell of 25 kg (Qi et al. 2024). The sourdough bread samples were cut into cubes (2 cm long x 2 cm wide, 1.5 cm thick). Acidity of SB formulations was evaluated by titration with NaOH (0.1 M) (AOAC 2019).

#### 2.2.7 Consumer Response Analysis

Sourdough bread samples were evaluated by 100 untrained consumers, 74% between 18 and 30 years old and 26% between 31 and 55 years old. The study was reviewed and approved by the Maringá State University (CAAE: 50306321.4.0000.0104), and informed consent was obtained from each subject prior to their participation in the study. The volunteers were recruited by invitations on social media. The sensorial test was performed in individual cabins, in four sessions. SBs were cut (4 × 2 × 2 cm) and served in cups coded with three‐digit random numbers and presented to the participants in a monadic manner. The overall acceptance assessment was carried out in terms of general acceptability, aroma, color, flavor, and texture, using a structured hedonic scale of nine points ranging from “1 – I extremely disliked it” to “9 – I extremely liked it.” The tasters were also asked about their intention to purchase the samples presented, using a structured five‐point scale that ranged from “1 – certainly would not buy” to “5 – certainly would buy” (Murray et al. 2001).

### 2.3 Statistical Analysis

The analyses were performed in triplicate, and the results were expressed as mean ± standard deviation. The data were subjected to analysis of variance (one‐way ANOVA) to compare the means following the Tukey test (*p* < 0.05), using the Sisvar program (version 5.3) (Ferreira 2019).

## 3 Results and Discussion

### 3.1 Chemical Composition of OPNP

The initial moisture of OPN leaves was 86.91 ± 0.39 g 100 g^−1^. After drying, the final moisture was 5.31 ± 0.39 g 100 g^−1^. Chemical composition (d.b.) is presented in Table 1. Protein content (21.01 g 100 g^−1^) was within the range (14.3–25.5 g 100 g^−1^) reported by Fioroto et al. (2024) and Barreira et al. (2020). The latter study also reported a similar lipid content (10.8 g 100 g^−1^) but a higher TDF content (41.8 g 100 g^−1^) than those found in the present study (Table 1). Notably, the TDF fraction of OPNP is composed of 80% insoluble fibers and 20% soluble fibers. Fiber intake is highly beneficial for health: IDFs normalize intestinal mobility, preventing constipation and diverticulitis, whereas SDFs are effective in reducing blood LDL cholesterol levels (Zhang et al. 2024).

**TABLE 1:** **C**omposition, instrumental color, and functional properties of ora‐pro‐nóbis leaf powder.

Parameters	Value
Proteins (g 100 g^−1^, d.b.)	21.1 ± 0.20 8.01 ± 0.56 19.66 ± 0.06 24.57 ± 1.78 6.35 ± 0.04 30.92 ± 1.74
Fats (g 100 g^−1^, d.b.)	
Ashes (g 100 g^−1^, d.b.)	
Insoluble dietary fiber (g 100 g^−1^, d.b.)	
Soluble dietary fiber (g 100 g^−1^, d.b.) Total dietary fiber (g 100 g^−1^, d.b.)	
Carbohydrates (g 100 g^−1^, d.b.)	18.42 ± 0.78
Ca (g 100 g^−1^, d.b.)	3.28 ± 0.04
Mg (g 100 g^−1^, d.b.)	1.10 ± 0.02
P (g 100 g^−1^, d.b.)	0.10 ± 0.01
K (g 100 g^−1^, d.b.)	3.07 ± 0.01
Na (mg 100 g^−1^, d.b.)	0.10 ± 0.00
Fe (mg 100 g^−1^, d.b.)	22.20 ± 2.63
Cu (mg 100 g^−1^, d.b.)	1.89 ± 0.08
Zn (mg 100 g^−1^, d.b.)	3.97 ± 0.26
Mn (mg 100 g^−1^, d.b.)	54.48 ± 2.61
L*	45.75 ± 0.02
a*	−3.54 ± 0.02
b*	26.23 ± 0.03
Water solubility index (g g^−1^, d.b.)	0.15 ± 0.02
Water absorption index (g g^−1^, d.b.)	6.72 ± 0.08
Oil absorption index (g g^−1^, d.b.)	2.08 ± 0.04

Abbreviation: d.b., dry basis.

Calcium was the most abundant mineral in OPNP (3.28 g 100 g^−1^), as previously reported by Sato et al. (2018). In addition to being crucial for bone health, calcium participates in numerous physiological functions, such as muscle contraction, blood coagulation, and immune response (Yang et al. 2024). The most important microelements present in OPNP were iron (22.20 g 100 g^−1^), manganese (54.48 g 100 g^−1^), and zinc (3.97 g 100 g^−1^). These results indicate that OPNP serves as an important mineral source and can be used to nutritionally enrich food products and culinary preparations.

### 3.2 Instrumental Color and Functional Properties of OPNP

Regarding color parameters (Table 1), OPNP had low lightness (L* > 50), with a greenish (negative a*) and yellowish (positive b*) color. The color parameters measured in the current study were similar to those found by Monteiro et al. (2021). These results indicate that incorporating OPNP into food products can change their color, producing either a desirable or an undesirable effect that should be evaluated by sensory analysis.

In relation to functional properties, the low WSI is correlated with the low contents of SDFs, sugars, and soluble proteins in OPNP. On the other hand, the WAI value of OPNP was higher than the values commonly found for conventional bread (1.5 to 2.1 g g^−1^) and wheat flour (3.43 g g^−1^), which can be attributed to its high contents of proteins and dietary fibers (Munshi and Dashora 2024). WSI is an important parameter when selecting ingredients for bakery products, as it contributes to dough handling, prevents drying during storage, and influences mouthfeel, consistency, and appearance (Okwunodulu et al. 2024). The OAI of OPNP was three times lower than its WAI, but it was within the range (1.12–2.30 g g^−1^) reported for different types of flour used in bread production (Munshi and Dashora 2024). An adequate OAI is essential for enhancing the flavor and mouthfeel of food products and contributes positively to the baking process (Okwunodulu et al. 2024).

### 3.3 Phenolic Compounds and Antioxidant Activity of OPNP

OPNP had a total phenolic content of 69.42 ± 1.43 mg GAE 100 g^−1^. A total of 13 phenolic compounds were identified, including 7 phenolic acids and 6 flavonoids (Table 2).

**TABLE 2:** Phenolic characterization of ora‐pro‐nóbis leaf powder.

Compound	Molecular formula	Real m/z [M‐H]^−^	Experimental m/z [M‐H]^−^	Rt (min)
Glucuronic acid	C_6_H_10_O_7_	193.0342	193.0346	0.82
Malic acid	C_4_H_6_O_5_	133.0131	133.0137	3.55
Citric acid	C_6_H_8_O_7_	191.0186	191.0190	3.58
Hydrobenzoic acid	C_7_H_6_O_3_	137.0233	137.0237	3.75
Caffeic acid	C_9_H_8_O_4_	179.0350	179.0340	3.92
Benzoic acid	C_7_H_6_O_2_	121.0280	121.0287	4.34
Ferulic acid	C_10_H_10_O_4_	193.0495	193.0496	4.04
Quercetin	C_15_H_10_O_7_	301.0348	301.0337	4.04
Quercetagetin	C_15_H_10_O_8_	317.0297	317.0261	3.98
Kaempferol	C_15_H_10_O_6_	285.0399	285.0387	4.19
Rutin	C_27_H_30_O_16 _	609.1455	609.1440	3.73
Isorhamnetin‐3‐O‐rutinoside	C_28_H_32_O_16_	623.1612	623.1591	3.73
Nicotiflorin	C_27_H_30_O_15 _	593.1506	593.1488	3.76

Abbreviations: m/z, mass‐to‐charge ratio; Rt, retention time.

Garcia et al. (2019) analyzed OPN and found 10 phenolic compounds, of which 2 were phenolic acids (caftaric and caffeic acids) and 8 were flavonoids (quercetin, kaempferol, and derivatives of the glycoside isorhamnetin). Cruz et al. (2021) detected 26 compounds in an OPN extract, 20 of which were glycosides other than quercetin, kaempferol, and isorhamnetin, in addition to caftaric, chicoric, caffeoylhexaric (2), and coumaroylhexaric (2) acids. Silva et al. (2024) found catechin, hydroxybenzoic acid, chlorogenic acid, caffeic acid, coumaric acid, ferulic acid, quercetin, *trans*‐cinnamic acid, and kaempferol in OPN flours. Some of these compounds were also detected in the present study. Of note, this is the first report of the presence of quercetagenin and glucuronic, malic, citric, and benzoic acids in OPNP. Phenolic composition can vary according to cultivation conditions, locality, harvesting methods, drying conditions, and extraction methods (Macedo et al. 2023).

Owing to their structure and ability to scavenge free radicals, phenolic compounds were associated with antioxidant activity. OPNP extract showed potential in stabilizing DPPH radicals (7.03 ± 0.24 mmol TE g^−1^) and reducing ferric in the FRAP assay (16.90 ± 0.28 mmol TE g^−1^). As occurs for other non‐conventional food products, there is limited information on the phenolic profile and antioxidant potential of OPNP. This study is relevant in light of the growing interest of food industries in natural sources of additives, such as antioxidants, which are used to extend shelf life, enhance flavor, and prevent oxidation in food products (Iqbal et al. 2022).

### 3.4 Lactic Acid Bacteria and Yeast Counts in the Leavening Agent

The leavening agent prepared in this study contained 7.40 log CFU g^−1^ LAB and 4.98 log CFU g^−1^ yeasts. Such a composition is similar to that reported by Stefanello et al. (2018), that is, a LAB concentration of 6.58 log CFU g^−1^ and a yeast concentration of 5.43 log CFU g^−1^. Wang et al. (2020) reported that the sourdough microbiota is generally characterized by higher levels of LAB than of yeasts. According to the authors, LAB produce organic acids that improve the rheological properties of dough and reduce the time needed for gluten development, which increases the flexibility of the gluten network. Moreover, LAB metabolites can improve sensory properties and enhance shelf life (Arora et al. 2021).

More than 70 bacterial species have been identified in sourdough leavening agents, the most common of which include Levilactobacillus brevis, Limosilactobacillus fermentum, Lactiplantibacillus plantarum, Companilactobacillus paralimentarius, and Fructilactobacillus sanfranciscensis (Lima et al. 2023). The leavening agent used here was characterized in a previous study and found to have L. brevis, Lactiplantibacillus pentosus, and Limosilactobacillus pontis as the predominant LAB and S. cerevisiae as the predominant yeast (Silva et al. 2025). Interestingly, this is the first report of bread production using this leavening agent in combination with OPNP. The following section presents the characterization of the experimental SB formulations.

### 3.5 Characterization of SB Formulations

#### 3.5.1 Chemical Composition and Antioxidant Properties

Table 3 shows the characteristics of SB formulations prepared using OPNP.

**TABLE 3:** Composition and antioxidant properties of sourdough breads produced with the addition of ora‐pro‐nóbis leaf powder (OPNP).

Parameters	SBC	SB1	SB3	SB5
Moisture (g 100 g^−1^)	39.95^c^ ± 0.24	40.41^c^ ± 0.11	41.08^b^ ±0.17	45.81^a^±0.13
Proteins (g 100 g^−1^, d.b.)	10.93^b^ ± 0.26	11.05^b^ ± 0.30	11.31^b^ ± 0.24	12.11^a^ ± 0.03
Fats (g 100 g^−1^, d.b.)	0.13^b^ ± 0.00	0.21^b^ ±0.02	0.70^a^ ± 0.02	0.71^a^ ± 0.03
Ashes (g 100 g^−1^, d.b.)	1.39^d^ ± 0.09	1.79^c^ ± 0.07	2.15^b^ ± 0.07	2.82^a^ ± 0.06
IDF (g 100 g^−1^, d.b.)	3.81^c^ ± 0.20	4.38^c^ ± 0.28	5.66^b^ ± 0.20	7.21^a^ ± 0.07
SDF (g 100 g^−1^, d.b.) TDF (g 100 g^−1^, d.b.)	1.12^a^ ± 0.09 4.93^d^ ± 0.11	1.26^a^ ± 0.13 5.64^c^ ± 0.15	1.34^a^ ± 0.07 7.00^b^ ± 0.22	1.41^a^ ± 0.12 8.62^a^ ± 0.09
Carbohydrates (g 100 g^−1^, d.b.)	82.45^a^ ± 0.30	81.42^a^ ± 0.45	78.69^b^ ± 0.54	75.71^c^ ± 0.08
Ca (g 100 g^−1^, d.b.)	0.04^d^ ± 0.01	0.12^c^ ± 0.00	0.24^b^ ± 0.00	0.34^a^ ± 0.00
Mg (g 100 g^−1^, d.b.)	0.07^d^ ± 0.00	0.09^c^ ± 0.00	0.13^b^ ± 0.00	0.16^a^ ± 0.00
P (g 100 g^−1^, d.b.)	0.17 ± 0.00	0.17 ± 0.00	0.17 ± 0.01	0.17 ± 0.00
K (g 100 g^−1^, d.b.)	0.05^c^ ± 0.01	0.06^c^ ± 0.00	0.09^b^ ± 0.00	0.11^a^ ± 0.01
Fe (mg 100 g^−1^, d.b.)	4.91 ± 0.17	4.68 ± 0.49	4.20 ± 0.77	4.70 ± 0.24
Cu (mg 100 g^−1^, d.b.)	0.40 ± 0.00	0.45 ± 0.05	0.35 ± 0.05	0.30 ± 0.00
Zn (mg 100 g^−1^, d.b.)	1.67 ± 0.11	2.06 ± 0.13	1.77 ± 0.06	1.85 ± 0.14
Mn (mg 100 g^−1^, d.b.)	2.48^b^ ± 0.09	2.87^b^ ± 0.38	3.64^ab^ ± 0.42	4.25^a^ ± 0.18
TPC (mg GAE 100 g^−1^, d.b.)	0.92^c^ ± 0.05	1.08^b^ ± 0.02	1.09^b^ ± 0.06	1.36^a^ ± 0.04
FRAP (mMol TE g^−1^, d.b.)	0.11^d^ ± 0.00	0.17^c^ ± 0.02	0.40^b^ ± 0.03	0.63^a^ ± 0.02
DPPH (mMol TE g^−1^, d.b.)	nd	nd	0.09^b^ ± 0.00	0.16^a^ ± 0.01

*Note*: The mean values (± standard deviation, *n* = 3) followed by the same letters (in the same row) do not differ statistically (*p* < 0.05) by the Tukey test.

Abbreviations: d.b., dry basis; GAE, gallic acid equivalent; IDF, insoluble dietary fiber; QE, quercetin equivalent; SB1, sourdough bread with 1% of OPNP; SB3, sourdough bread with 3% of OPNP; SB5, sourdough bread with 5% of OPNP; SBC, sourdough bread control (without OPNP addition); SDF, soluble dietary fiber; TDF, total dietary fiber; TE, Trolox equivalent; TPC, total phenolic content.

SB5 exhibited the highest moisture content (45.81 g 100 g^−1^), followed by SB3 (*p* < 0.05). This finding indicates that the addition of ≥3% OPNP favors water retention in the final product. There was an increase (10%) in protein content in SB5 in relation to the control. Lipid content was higher in SB3 and SB5 than in SB1 and SBC. It should be noted that lipid contents ranged from 0.13 to 0.71 g 100 g^−1^ (d.b.), corresponding to the lowest values observed in proximate composition.

The TDF of formulations followed the descending order SB5> SB3> SB1> SBC. Additionally, there was an increase of 49% (SB3) and 90% (SB5) in IDF compared with SBC, which did not differ from SB1 (*p* > 0.05). SDF levels were similar among SB formulations.

Bakery goods are sources of carbohydrates, owing to the high amount of wheat flour in formulations. Here, carbohydrate content decreased by 5% and 9% in SB3 and SB5, respectively, compared with the other formulations. Such a finding may be attributed to the high TDF content of OPNP.

Regarding mineral composition, the contents of P, Fe, Cu, and Zn did not differ between SB formulations. The addition of 3% and 5% OPNP led to an increase in K and Mn contents compared with SBC and SB1. Ca and Mg contents increased proportionally to OPNP concentration. Compared with SBC, SB5 had a 750% higher Ca content and a 129% higher Mg content. Sato et al. (2018) observed a gradual increase in Ca content (0.05%, 0.41%, and 1.18%) in spaghetti formulations added with 0, 10%, and 20% OPN flour.

There was an increase in total phenolic content with increasing concentration of OPNP. SB5 had a 48% higher total phenolic content than SBC. Similarly, a 5% addition of *Opuntia ficus‐indica* cladodes in bread formulations resulted in a 6‐fold increase in total phenolic content (Msaddak et al. 2017).

Phenolic compounds may act as antioxidants, explaining the increase in the FRAP antioxidant activity of SB formulations with OPNP addition. By the DPPH method, antioxidant activity was detected only in SB3 and SB5. The increase in OPNP addition from 3% to 5% resulted in a 78% increase in DPPH scavenging activity. Bavaro et al. (2025) also reported an increase in antioxidant activity in functional breads enriched with artichoke bract powder. According to the authors, antioxidant compounds were found in considerable quantities after baking, underscoring the importance of adding functional ingredients to bakery products.

#### 3.5.2 Instrumental Color, Texture Profile, and Acidity

Table 4 shows the results of instrumental color, texture, and acidity analyses of the SB formulations prepared in this study.

**TABLE 4:** Instrumental color, acidity, and textural properties of sourdough breads produced with the addition of ora‐pro‐nóbis leaf powder (OPNP).

Bread	Parameters	SBC	SB1	SB3	SB5
Crumb	L*	57.27^a^ ± 0.95	40.42^b^ ± 0.22	30.10^c^ ± 0.64	25.52^d^ ± 0.37
	a*	1.63^a^ ± 0.10	−2.73^b^ ± 0.07	−2.75^b^ ± 0.11	−2.62^b^ ± 0.07
	b*	20.17^a^ ± 1.99	19.85^a^ ± 0.57	19.76^a^ ± 0.29	16.59^b^ ± 0.59
Lateral crust	L*	61.92^a^ ± 0.90	52.61^b^ ± 1.50	45.91^c^ ± 1.40	41.57^d^ ± 2.11
	a*	5.04^a^ ± 0.28	0.88^b^ ± 0.03	−0.46^c^ ± 0.02	−1.31^d^ ± 0.07
	b*	23.86^a^ ± 1.97	17.21^b^ ± 1.75	12.81^c^ ± 1.22	9.79^c^ ± 0.70
Central crust	L*	63.57^a^ ± 1.72	44.26^b^ ± 1.08	38.62^c^ ± 0.44	28.82^d^ ± 2.90
	a*	3.56^a^ ± 0.36	1.80^b^ ± 0.14	−1.56^c^ ± 0.11	−1.80^c^ ± 0.09
	b*	24.39^a^ ± 1.04	21.46^b^ ± 0.54	19.61^c^ ± 0.98	17.76^d^ ± 0.51
—	Acidity (%)	1.33^c^ ± 0.03	1.35^c^ ± 0.00	1.63^b^ ± 0.01	1.84^a^ ± 0.01
	Hardness (N)	7.49 ± 0.47	7.36 ± 0.66	7.07 ± 0.91	7.99 ± 0.64
	Elasticity	0.75 ± 0.01	0.87 ± 0.04	0.81 ± 0.06	0.84 ± 0.06
	Cohesiveness	0.74^a^ ± 0.03	0.72^a^ ± 0.03	0.66^b^ ± 0.01	0.72^ab^ ± 0.04
	Gumminess (N)	6.57^a^ ± 0.62	5.31^b^ ± 0.48	4.29^c^ ± 0.34	5.42^b^ ± 0.56
	Chewability (N)	5.02 ± 0.60	4.73 ± 0.26	4.29 ± 0.32	4.86 ± 0.31

*Note*: The mean values (± standard deviation, *n* = 3) followed by the same letters (in the same row) do not differ statistically (*p* < 0.05) by the Tukey test.

Abbreviations: a*, red/green coordinate; b*, yellow/blue coordinate; L*, luminosity; SB1, sourdough bread with 1% of OPNP; SB3, sourdough bread with 3% of OPNP; SB5, sourdough bread with 5% of OPNP; SBC, sourdough bread control (without OPNP addition).

**FIGURE 1:**
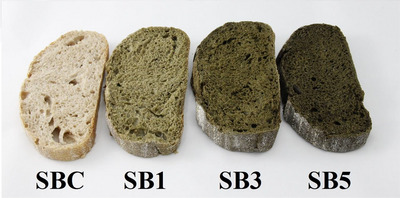
Formulations of sourdough breads with the addition of ora‐pro‐nóbis leaf powder. SB1, sourdough bread with 1% of OPNP; SB3, sourdough bread with 3% of OPNP; SB5, sourdough bread with 5% of OPNP.

L* values decreased proportionally to increasing OPNP levels, representing a darkening of SBs in both the crumb and crust. Furthermore, OPNP addition led to a decrease in a* and b* values in SB crusts, indicating higher greenness and lower yellowness, respectively. For the crumb, a* values were lower in OPNP‐added formulations than in SBC, whereas b* values did not differ between SBC, SB1, or SB3, which had higher values than SB5. Sato et al. (2018) found that OPN flour addition to spaghetti formulations resulted in product darkening, with a* values not differing between formulations containing 10% and 20% OPN flour. The color of SB formulations prepared in this study can be observed in Figure 1.

OPNP addition to SBs did not influence hardness, elasticity, or chewability. These results are interesting, given that an increase in hardness and elasticity is negatively correlated with bread quality (Cao et al. 2023). SB3 had a lower cohesiveness than SBC and SB1. Gumminess also decreased with OPNP addition (SB1, SB3, and SB5). A similar effect was reported by Waters et al. (2012) for bread formulated with fermented brewer's spent grain compared to control breads (refined and whole wheat flour). Gumminess reduction may be related to the increase in SDF levels in OPNP‐added SBs (Table 4). Dietary fibers weaken the gluten network in bread dough; however, soluble fibers can improve water retention, owing to their high level of hydrophilic compounds, improving textural properties (Bavaro et al. 2025). The reduction in cohesiveness and gumminess can be considered positive for consumers, as these properties influence overall mouthfeel during mastication (Amoah et al. 2020).

The addition of 3% or 5% OPNP increased the acidity of SB formulations compared with SBC and SB1. This effect may be due to the presence of soluble carbohydrates in OPNP, which are susceptible to fermentation by LAB present in the leavening agent (Bartkiene et al. 2013). Acidity favors the preservation of SBs by inhibiting the development of pathogenic microorganisms (Lafuente et al. 2024).

#### 3.5.3 Consumer Acceptance

The results of the sensory acceptance assay revealed a decrease in mean scores with OPNP addition (Table 5). Similarly, Msaddak et al. (2017) reported that the addition of *O. ficus‐indica* cladodes caused a reduction in the sensory acceptance of bread formulations. Peñalver and Nieto (2024) also observed that gluten‐free breads enriched with spirulina caused a decrease in all sensory attributes compared to control breads.

**TABLE 5:** Sensory acceptance of sourdough breads produced with the addition of ora‐pro‐nóbis leaf powder (OPNP).

Formulations	SBC	SB1	SB3	SB5
Color	8.00^a^ ± 0.92	6.11^b^ ±1.47	5.59^c^ ± 1.74	5.15^c^ ± 1.89
Aroma	6.93^a^ ± 1.41	6.02^b^ ± 1.42	5.95^b^ ± 1.70	5.66^b^ ± 1.62
Flavor	6.40^a^ ± 1.85	5.75^b^ ± 1.87	5.77^b^ ± 1.99	5.28^b^ ± 1.87
Texture	7.29^a^ ± 1.23	6.77^b^ ± 1.51	6.55^b^ ± 1.57	6.54^b^ ± 1.60
Overall acceptance	6.70^a^ ± 1.66	5.96^b^ ± 1.61	5.94^b^ ± 1.63	5.40^c^ ± 1.62

*Note*: The mean values (± standard deviation, *n* = 100) followed by the same letters (in the same row) do not differ statistically (*p* < 0.05) by the Tukey test.

Abbreviations: SB1, sourdough bread with 1% of OPNP; SB3, sourdough bread with 3% of OPNP; SB5, sourdough bread with 5% of OPNP.

Regarding color, SB1 scored higher than SB3 and SB5, attributed to the greenish color conferred by OPNP addition at higher levels. Bavaro et al. (2025) reported that increasing the amount of artichoke bract powder in enriched functional breads resulted in significant changes to texture and color, particularly in breads enriched with 15% artichoke powder. Nurtiana et al. (2023) observed that the increase in dietary fiber and pigment, caused by the addition of beneng flour and rice bran to breads, decreased the sensory acceptance of products. Overall, these findings are in line with those observed in the current study.

Aroma, flavor, and texture did not differ between SB formulations added with OPNP (SB1, SB3, and SB5). Additionally, overall liking scores were similar between SB1 and SB3 and lower in SB5. Lafarga et al. (2019) reported that texture, overall liking, and appearance were not affected by a 2% addition of broccoli to bread. Dziki et al. (2014) stated that consumer satisfaction can be achieved when the replacement of wheat flour by plant‐based ingredients occurs in percentages up to 5%. These findings corroborate the results of this study, in that OPNP addition at lower levels minimizes the changes to sensory attributes.

**FIGURE 2:**
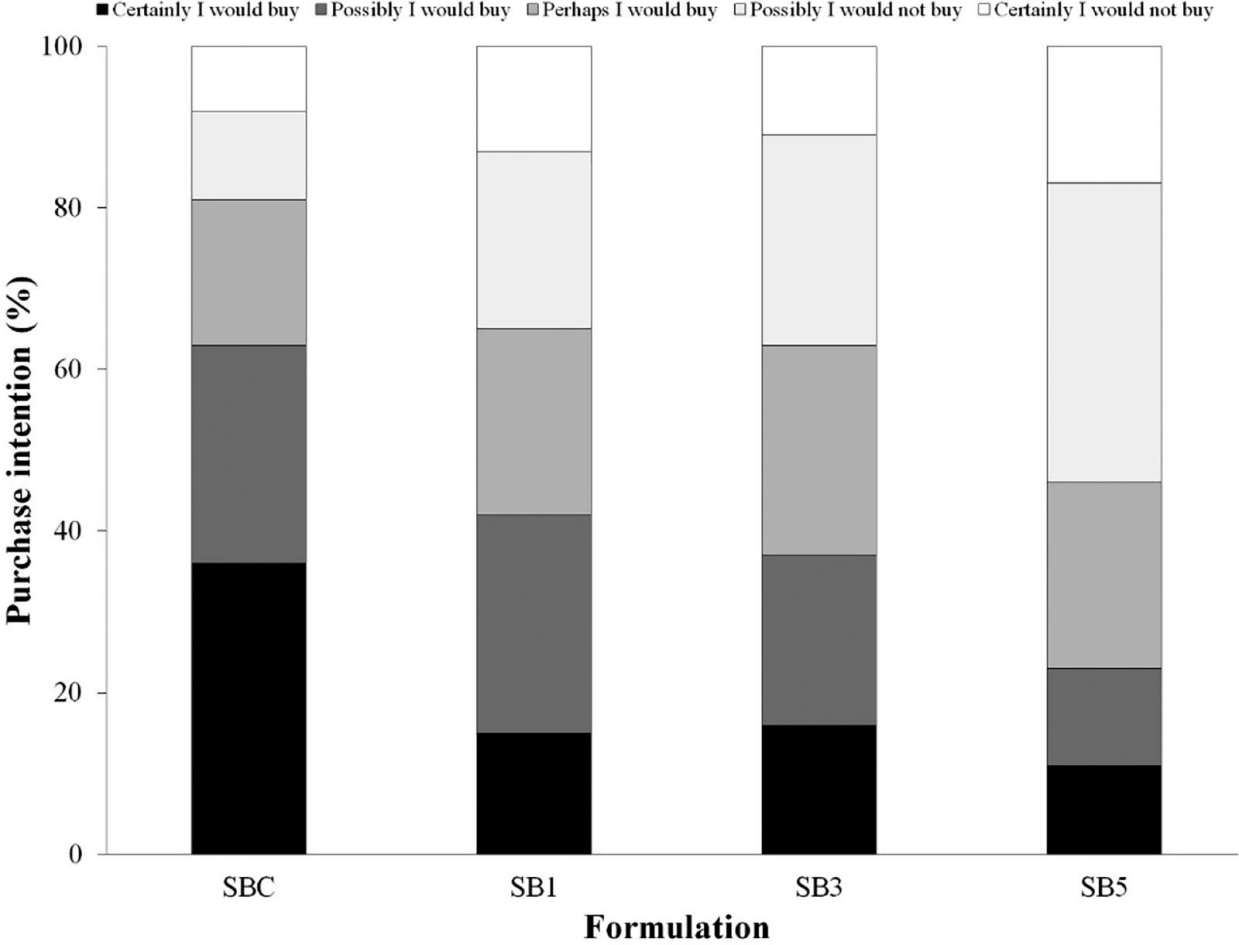
Purchase intention of sourdough bread formulations with the addition of ora‐pro‐nóbis leaf powder. SB1, sourdough bread with 1% of OPNP; SB3, sourdough bread with 3% of OPNP; SB5, sourdough bread with 5% of OPNP.

Panelists' purchase intention (Figure 2) toward SBC was as follows: 36% definitely would buy, 27% probably would buy, and 18% might or might not buy. SB1 and SB3 had similar results, with ∼15% of panelists selecting definitely buy, ∼24% probably buy, and ∼24% might or might not buy. For SB5, the panelists reported that they would definitely buy (11%), probably buy (12%), or might or might not buy (23%). Notably, 54% of tasters selected probably or definitely not buy, underscoring the low sensory acceptance of this formulation (Table 5).

Neophobic behaviors likely contributed to the lower sensory scores for OPNP‐added formulations, as greenish breads are rarely encountered commercially (Atzler et al. 2021). Previous studies have reported that neophobia can negatively impact consumer ratings of novel foods in overall liking tests (Park and Cho 2016; Andrade et al. 2015; Franceschinis et al. 2025). Moreover, the low sensory acceptance scores can be associated with the low consumption of SBs by panelists: 52% of panelists never consumed SBs, 34% consume SBs once a month, 8% consume SBs once a week, 4% consume SBs two to three times a week, and only 2% consume SBs daily. Most tasters had the habit of consuming quick‐fermentation breads with neutral aromas and flavors, unlike SBs, which are known for their slightly acidic flavor. This fact may have negatively influenced panelists' ratings of the different attributes.

The sensory panel consisted predominantly of young consumers, with 74% aged between 18 and 30 years. This demographic profile may have influenced the sensory evaluation results, as young individuals tend to be more open to novel foods but can also display stronger neophobic reactions when faced with unfamiliar colors or flavors (Atzler et al. 2021; Park and Cho 2016). Consumer segmentation has been recognized as a critical factor in the acceptance of functional and non‐conventional food products, with variables such as age, lifestyle, and SB familiarity strongly affecting perception. For instance, older consumers may value more nutritional benefits and fiber enrichment, whereas younger consumers may be more sensitive to deviations in appearance and flavor from conventional products (Baker et al. 2022; Safraid et al. 2024). Therefore, the predominance of young panelists in this study may have biased the results toward a lower acceptance of the greenish color and acidic notes conferred by OPNP addition. Future studies should consider a more balanced demographic distribution to better understand how different consumer segments perceive and value OPNP‐enriched breads.

## 4 Conclusion

OPNP addition contributed to increasing the contents of dietary fibers, calcium, magnesium, and antioxidant compounds in SB formulations. SB5 had the highest protein content and lowest carbohydrate content, differing from the control. Hardness, elasticity, and chewability did not differ among formulations. Instrumental color parameters L*, a*, and b* were modified by OPNP addition, negatively impacting sensory acceptance. Similar values of aroma, flavor, and texture were observed for SB1, SB3, and SB5, but SB1 and SB3 achieved higher overall acceptance and better purchase intention. This study contributes to the literature by demonstrating the benefits of SB production with OPNP as a low‐cost nutrient source.

## Author Contributions

**Dayane Lilian Gallani Silva**: resources, methodology, investigation, formal analysis, data curation, writing – review and editing, writing – original draft. **Filipe Andrich**: conceptualization, writing – review and editing, writing – original draft. **Barbara Daniele Almeida Porciuncula**: formal analysis, writing – review and editing. **Marcelo Augusto Batista**: methodology, formal analysis, writing – review and editing. **Marcela Moreira Terhaag**: methodology, supervision, formal analysis, writing – review and editing. **Beatriz Cervejeira Bolanho Barros**: funding acquisition, writing – review and editing, writing – original draft, visualization, validation, supervision, project administration, conceptualization.

## Conflicts of Interest

The authors declare no conflicts of interest.

## Supporting information




**Supplementary Table**: jfds70629‐sup‐0001‐table S1.docx

## Data Availability

The data presented in the figures of this study are available upon request from the corresponding author.
