# Supplementary material for: Nutritional Profile and Sensory Acceptance of Sourdough Breads Enriched With Ora‐pro‐nóbis Leaf Powder
Source: J Food Sci. 2025 Oct 24;90(10):e70629. doi: 10.1111/1750-3841.70629 (PMC12552800; doi:10.1111/1750-3841.70629)
Supplement: Supplementary file 1 — Supplementary Table: jfds70629‐sup‐0001‐table S1.docx [file JFDS-90-0-s001.docx]

**Table 1**S

Formulations of sourdough breads produced with the addition of ora-pro-nóbis leaf powder (OPNP)

| Ingredients (%) | SBC | SB1 | SB3 | SB5 |
| --- | --- | --- | --- | --- |
| Wheat flour | 23.43 | 22.93 | 21.93 | 20.93 |
| Whole wheat flour | 23.43 | 22.93 | 21.93 | 20.93 |
| Levain | 18.89 | 18.89 | 18.89 | 18.89 |
| Water | 32.21 | 32.21 | 32.21 | 32.21 |
| Sucrose | 1.02 | 1.02 | 1.02 | 1.02 |
| Salt | 1.02 | 1.02 | 1.02 | 1.02 |
| OPNP | 0 | 1 | 3 | 5 |

SBC – sourdough bread control (without OPNP addition), SB1 - sourdough bread with 1% of OPNP, SB3 - sourdough bread with 3% of OPNP, SB5 - sourdough bread with 5% of OPNP.
